# Detection of Image Artifacts Using Improved Cascade Region-Based CNN for Quality Assessment of Endoscopic Images

**DOI:** 10.3390/bioengineering10111288

**Published:** 2023-11-06

**Authors:** Wei Sun, Peng Li, Yan Liang, Yadong Feng, Lingxiao Zhao

**Affiliations:** 1 School of Biomedical Engineering (Suzhou), Division of Life Sciences and Medicine, University of Science and Technology of China, Hefei 230026, China; sunw@sibet.ac.cn; 2 Suzhou Institute of Biomedical Engineering and Technology, Chinese Academy of Sciences, Suzhou 215163, China; lipeng@sibet.ac.cn; 3 Department of Gastroenterology, Zhongda Hospital, School of Medicine, Southeast University, 87 Dingjiaqiao Road, Nanjing 210009, China; ly4235@163.com (Y.L.); drfengyd@126.com (Y.F.)

**Keywords:** endoscopic image quality assessment, endoscopy artifacts, convolutional neural network

## Abstract

Endoscopy is a commonly used clinical method for gastrointestinal disorders. However, the complexity of the gastrointestinal environment can lead to artifacts. Consequently, the artifacts affect the visual perception of images captured during endoscopic examinations. Existing methods to assess image quality with no reference display limitations: some are artifact-specific, while others are poorly interpretable. This study presents an improved cascade region-based convolutional neural network (CNN) for detecting gastrointestinal artifacts to quantitatively assess the quality of endoscopic images. This method detects eight artifacts in endoscopic images and provides their localization, classification, and confidence scores; these scores represent image quality assessment results. The artifact detection component of this method enhances the feature pyramid structure, incorporates the channel attention mechanism into the feature extraction process, and combines shallow and deep features to improve the utilization of spatial information. The detection results are further used for image quality assessment. Experimental results using white light imaging, narrow-band imaging, and iodine-stained images demonstrate that the proposed artifact detection method achieved the highest average precision (62.4% at a 50% IOU threshold). Compared to the typical networks, the accuracy of this algorithm is improved. Furthermore, three clinicians validated that the proposed image quality assessment method based on the object detection of endoscopy artifacts achieves a correlation coefficient of 60.71%.

## 1. Introduction

An endoscope—the most direct examination device for gastrointestinal diseases—is introduced into the human body through natural cavities. This has significant advantages in both diagnosis and treatment, and it serves as a primary means for subsequent minimally invasive surgery and noninvasive treatment [1]. The collected endoscopic images are used to determine the patient’s condition and formulate subsequent treatment plans. However, endoscopic images contain numerous interfering factors, such as mirror reflection, motion blur, bubbles, etc., which can affect the visual interpretation of endoscopic examinations, affect the observation and diagnosis of clinicians regarding the lesion area, and negatively affect computer-aided diagnosis (CAD) processes [2]. In addition, the presence of interference is an important reference criterion for the assessment of image quality in gastrointestinal endoscopy images, contributing to the quality evaluation of clinical endoscopic examination procedures [3].

Image quality assessment aims to simulate human perception and is typically performed by human observers who evaluate algorithm values against subjective ratings. A typical approach involves comparing the distortion metric with an ideal imaging model or a perfect reference image [4]. Depending on the amount of information provided by the original reference image, it can be classified as full, reduced, or no-reference (also known as blind image quality assessment, BIQA) [5]. No-reference image quality assessment (NR-IQA) is one of the most challenging problems in image quality assessment because it solely relies on distorted images, and thus has garnered significant research interest in recent years.

NR-IQA has a significant practical value and is widely used in numerous practical applications where reference images are unavailable. Mittal et al. proposed a blind image spatial quality assessment method called the blind/referenceless image spatial quality evaluator (BRISQUE) [6]. This method uses locally normalized luminance coefficients to quantify the “naturalness” loss caused by distortion; it exhibits a low computational complexity, making it suitable for real-time applications. Liu et al. introduced RankIQA [7], which generates ordered degraded images to train a Siamese network for relative quality ranking. The trained network is then transferred to a traditional CNN, enabling it to estimate the absolute image quality from a single image. Junjie et al. proposed a multiscale image quality transformer called MUSIQ [8], which can handle full-resolution image inputs with different resolutions, sizes, and aspect ratios; capture image quality at various granularities; and perform well on multiple large-scale IQA datasets. Talebi et al. presented a neural network based on deep object recognition called NIMA [9], which can predict the distribution of human evaluations of images in terms of both perceptual quality (from a technical standpoint) and attractiveness (from an aesthetic standpoint). The proposed neural network exhibited scores that closely resembled the human subjective ratings, making it suitable for natural image quality assessment tasks. With the profound influence of machine learning, particularly deep learning, in various fields, image quality assessment in endoscopy is undergoing continuous innovation. Alexander et al. developed a new fidelity score for quantitative image quality assessment based on the structural similarity maps adopted in the human visual system (HVS), where the measure indicated the extent to which the structural information of relevant structures was preserved in the panorama [10]. Aubreville et al. [11] proposed an improved version of the Inception V3 network for detecting motion artifacts in endoscopic images. Kamen et al. [12] selected high-quality and information-rich images by calculating the image entropy. Outtas et al. [13] studied the usability of two general algorithms based on natural image quality assessments (NIQE [14] and BRISQUE [6]) in medical image environments. Prerna et al. [15] proposed the use of gray-level co-occurrence matrices for image quality assessment. Zhang et al. [16] performed a no-reference quality assessment of capsule endoscopy images by calculating the gradient field using the Sobel operator.

**Figure 1:**
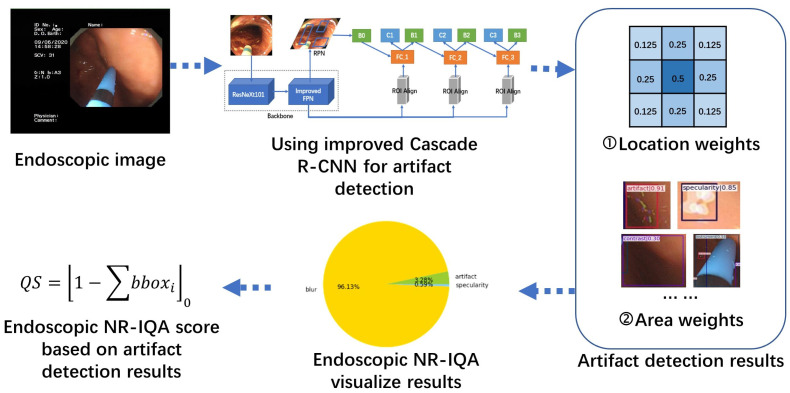
Architecture of the proposed framework for detection of image artifacts using improved cascade region-based CNN for no-reference endoscopic image quality assessment.

Considering the limitations of current no-reference endoscopic image quality assessment methods, which primarily focus on evaluating individual artifacts and often yield less interpretable results, we propose a framework for detecting endoscopic image artifacts and assessing image quality based on an improved cascade R-CNN designed to overcome these challenges. The architecture of the framework is shown in Figure 1. First, the original endoscopic image is obtained. After undergoing image preprocessing, the image is fed into the improved cascade region-based CNN for artifact detection. Subsequently, after extracting the detection result data, both location and area information are acquired and transmitted to the endoscopic image NR-IQA method. This process culminates in the generation of interpretable image quality assessment results.

The artifact detection method retains the multistage structure of the cascade R-CNN, with ResNeXt101 serving as the backbone network. We improved the original feature pyramid network (FPN) structure and introduced Generalized Intersection over Union (GIoU) loss as a new evaluation metric loss function, replacing the original IoU metric. Using the improved model, we successfully located and identified artifacts in the gastrointestinal tract. Additionally, we propose a method that combines multiple weights to calculate the image quality score based on artifact detection results.

The main contributions of this study are as follows:

(1) Nonvideo sequences of gastrointestinal endoscopy images from multiple clinical hospitals were used. The images have high content and modality diversity, including images from white light imaging (WLI), narrow-band imaging (NBI), and iodine staining, making them robust and relevant for general applications in gastrointestinal endoscopy.

(2) An improved feature pyramid network that incorporates channel attention mechanisms into the feature extraction process is proposed. Shallow and deep features were fused by introducing an additional channel from the shallow to deep layers, thereby enhancing the utilization of spatial information in the shallow layers. This not only strengthened the feature extraction of semantic and positional information through path aggregation but also established global spatial feature attention for the mapping and representation of artifact features across multiple branches in the image.

(3) The proposed endoscopic image quality assessment method successfully detects anomalies in small targets and handles multiple interferences. Moreover, it uses the detection results of high-quality artifact targets to provide numerical scores that are close to expert ratings, thereby demonstrating strong interpretability.

Overall, these contributions enhance the detection and assessment of endoscopic image artifacts and provide valuable insights for clinical multicenter endoscopic image quality assessment.

## 2. Materials

### 2.1. Data Acquisition

The endoscopic image data used in this study were obtained from 12 medical institutions in Jiangsu Province, with Zhongda Hospital Southeast University serving as the central hospital. In total, 2303 images containing artifacts were collected through data collection and clinicians’ annotations. The dataset encompassed images of various tissues and organs, including the esophagus and stomach, as well as multimodal endoscopic images (including WLI, NBI, and iodine-stained images), as shown in Table 1. The color space of the images was distributed as RGB, and eight types of interferences were present: blur, bubbles, specularity, saturation, contrast, instrument, blood, and artifacts, as listed in Table 2.

Specularity is caused by the reflection of the tube owing to the smooth surface of the electron endoscope; saturation and contrast are derived from light exposure and changes in videoscope topology; blur results from erratic hand movements and small movements of the digestive tract lining; and bubbles and blood result from changes in the digestive tract environment leading to the presence of fluids. This instrument, however, prevents the occlusion of the inner wall of the digestive tract. These artifacts originate from unpredictable imaging conditions, and they are present in more than 60% of endoscopic video frames [5].

For the image quality assessment, 100 images were randomly selected from the 2303 images containing artifacts and subjectively scored based on the evaluation criteria shown in Table 3.

### 2.2. Data Preprocessing

**Figure 2:**
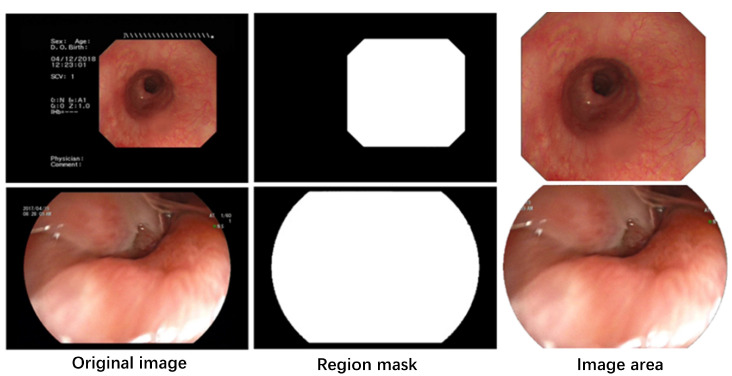
Endoscopic image preprocessing operations.

Significant differences exist between the sizes and types of endoscopic images captured by various models of digestive endoscopic instruments from various manufacturers. These images also contain nonendoscopic information such as instrument details and acquisition time. To mitigate the impact of these factors on the image quality assessment, this study employed dilation and erosion morphological processing methods to generate effective area masks. These masks were used to extract the relevant image regions, thereby enhancing the effectiveness of the endoscopic image quality assessment. The image preprocessing operation is illustrated in Figure 2.

(1) $$\begin{array}{c} \mathrm{CLOSE}(X)=E(D(X)) \end{array}$$

### 2.3. Data Analysis

**Figure 3:**
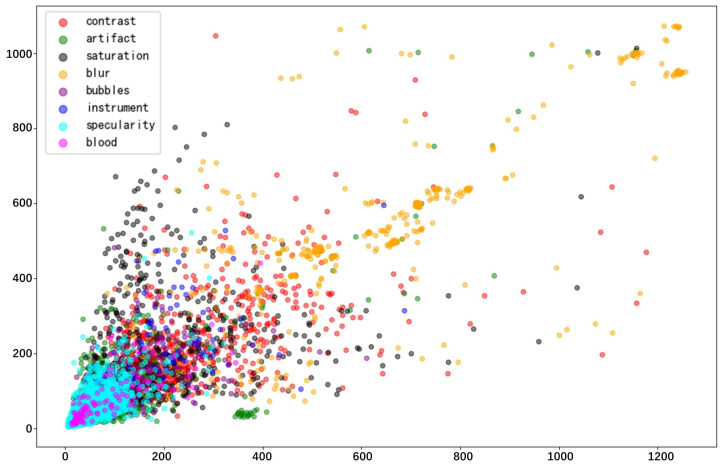
Analysis of statistical characteristics of sample data. The scale distribution of each defect category is shown, where the *x*-axis represents the height of the ground truth (GT) and the *y*-axis represents the width of the GT.

Upon analyzing the surface characteristics of the samples, substantial within-class variations and interclass correlations were observed among the artifact samples. For example, specularity and bubbles exhibit similarities in certain textural representations; however, specularity, bubbles, and artifacts follow distinct internal morphological patterns. The annotations were subjected to statistical analysis and the results are shown in Figure 3. Among the eight defined defect categories, the maximum difference in quantity approaches 64.5:1. Owing to the sample characteristics, the detection task encompassed highly imbalanced categories, extreme proportions, and small sample sizes.

### 2.4. Data Augmentation

The dataset used in this study was obtained from various devices across multiple hospitals, resulting in significant image resolution variations, such as 512×512, 768×576, 1081×808, 1349×1079, and 1920×1080. To facilitate the algorithmic processing, this section standardizes the image size to 800×800.

In addition to the dataset size and labels, certain characteristics of images and labels render the noise detection model less effective. Insufficiently qualified images with accurate annotations and imbalanced class distributions are common factors that lead to overfitting and limited generalization in neural network training. If the sample size for any of these eight types of defects is inadequate or significantly different, the trained model may exhibit a significant bias.

**Figure 4:**
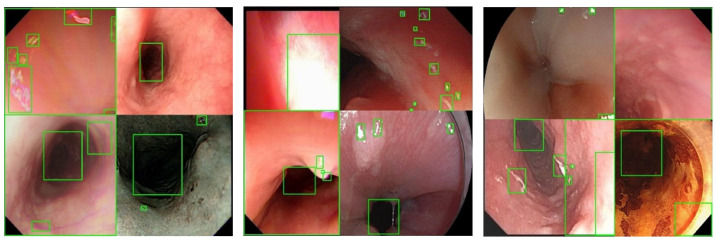
Performance of mosaics in data augmentation.

To address this issue, data augmentation techniques were employed in this study to alleviate the sample imbalance. Data augmentation involves the generation of variations in the original images, thereby increasing the training data volume without introducing additional images. Four types of geometric transformations are applied during the data augmentation process: mosaics [17], rescaling, inversion, and rotation. The results of the mosaic data enhancement are shown in Figure 4.

## 3. Methods

The overall approach of this method is divided into two main parts: artifact target detection using the improved cascade R-CNN model and endoscopic image quality assessment based on the detection results.

### 3.1. Cascade R-CNN

The cascade R-CNN [18] is an extension of the Faster R-CNN that aims to incorporate more semantic information into the detection task [19]. Unlike the traditional Faster R-CNN, the cascaded R-CNN employs a cascaded modular structure, enabling additional context and features to be extracted through advanced feature extraction steps. These features were then utilized in the ROI pooling layer to enhance the network performance by providing richer representations. In addition, the cascaded structure provides more supervised signals during training, leading to more accurate models.

The cascade R-CNN consists of a feature extraction network, an FPN, a region proposal network layer, and cascade detectors. The feature extraction network ResNeXt101 was used to extract image features. The original map is obtained by convolution operations, Conv1, Conv2, Conv3, Conv4, and Conv5, and feature fusion at different levels to obtain feature maps P2, P3, P4, and P5 at different scales. These feature maps were then input into the region proposal network to obtain the candidate target areas. Subsequently, ROI alignment operation was performed on the resulting candidate target areas to obtain an ROI feature map.

**Figure 5:**
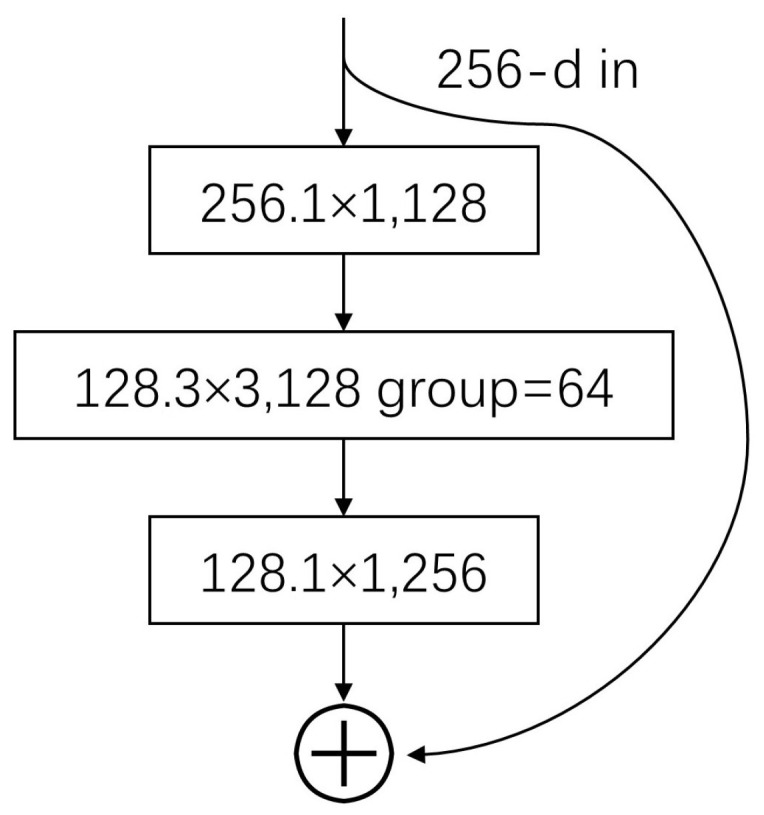
Residual structure.

ResNeXt101 is a deep CNN built on the ResNet architecture [20]. This introduces the concept of ”group convolution,” where multiple parallel convolutional branches with the same structure are employed. Each branch processes different input characteristics, enabling an increased network width without overfitting. The ResNeXt101 network incorporates techniques, such as batch normalization and residual connections, to enhance its performance. Residual structures have been widely employed in the field of medical imaging to eliminate dependencies between the network’s weak and high levels [21]. The residual structure is shown in Figure 5.

**Figure 6:**
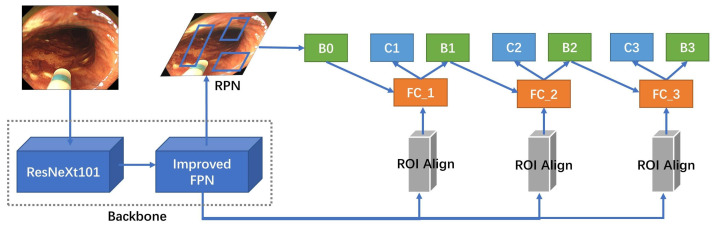
Artifact detection model based on the improved cascade R-CNN.

In this study, an improved cascade R-CNN detection model is proposed, utilizing ResNeXt101 as the backbone network to locate and identify artifacts in the digestive tract. The original evaluation index, namely Intersection over Union (IoU), in the cascade R-CNN was replaced with GIoU loss as a new evaluation index loss function. IoU measures the degree of overlap between a predicted bounding box and a ground truth bounding box. GIoU is an improved version of IoU. It not only considers the intersection and union areas but also takes into account the spatial relationship between the two bounding boxes, which makes it more suitable for complex object shapes and rotations. This enhancement is aimed at making it more robust to variations in object rotation and shape. Figure 6 illustrates the enhanced FPN with added attention mechanisms that enable better learning capabilities.

The advantages of this model are as follows:

(1) It provides an effective feature extraction structure that maximizes the utilization of shallow feature information and enhances the detection of small targets.

(2) The incorporation of the channel attention mechanism captures the feature dependencies between different channel maps in the feature extraction network. This reduces the missed detection rate and leads to more reliable results.

(3) GIoU was employed as the new evaluation index loss function, replacing IoU, the original evaluation index in cascade R-CNN. This ensures scale invariance in the loss function target detection frame regression and maintains consistency between the optimization objective and loss function of the detection frame.

### 3.2. Improved Feature Pyramid Structure

Each layer of the feature pyramid employs a distinct convolution kernel size to extract the features. The lower layers capture large-scale features, such as edges, whereas the final layer captures fine details. These feature maps are then concatenated to create a comprehensive feature map for classification. The key advantage of a feature pyramid is its ability to capture features at various scales in an image, resulting in enhanced classification performance. This is particularly effective for handling images of different sizes by encompassing the features of diverse scales.

Although this approach exhibits a high accuracy in classifying and localizing larger objects, it faces challenges in accurately detecting smaller objects. This is due to the nature of the deep CNN, where extensive convolution and pooling operations result in expanded receptive fields and decreased resolutions in the network feature layers. Consequently, there is the risk of overlooking small targets.

In contrast, low-level features obtained from shallow neural networks have a higher resolution and contain more information, making them valuable for detecting small objects. By fusing features at different scales, the recognition accuracy for small targets can be improved while maintaining the accuracy for larger targets.

**Figure 7:**
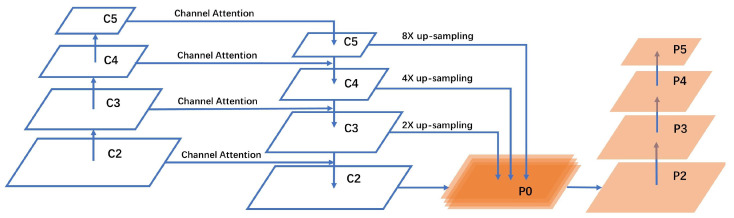
Improved feature pyramid structure.

The feature enhancement method was employed in this study to fully use shallow feature information and increase the resolution of the feature maps for small targets. Deep features, which contain rich semantic information, were added elementwise to shallow features through bilinear interpolation. However, an accurate localization of shallow to deep features has become increasingly challenging. To address this issue, an additional pathway, called the pathway enhancement channel, was introduced. The number of convolution layers traversed by the information flow from deep to shallow layers is reduced by this pathway, enabling the propagation of shallow information to deep layers and enhancing the localization of deep positional information. The improved structure of the FPN is shown in Figure 7.

The original convolutional output layers in the bottom-up pathway are $C_{2}$, $C_{3}$, $C_{4}$, and $C_{5}$. First, convolution was applied to the input image, followed by dimensionality reduction of $C_{3}$, $C_{4}$, and $C_{5}$. After applying the attention mechanism to $C_{5}$, it underwent a two-fold upsampling to match the size of $C_{4}$. Subsequently, the corresponding elements were added, and the obtained result was input into $C_{4}$. The same process was used to obtain $C_{3}$ and $C_{2}$. $C_{3}$, $C_{4}$, and $C_{5}$ were upsampled by 2x, 4x, and 8x, respectively, and added to the shallow feature maps using bilinear interpolation. Corresponding elements were added to increase the utilization of deep features for shallow features. The formula for bilinear interpolation f(x,y) is as follows:

(2) $$\begin{array}{c} \;\begin{array}{cc} \; & f\left(x,y\right)=\frac{f\left(x_{1},y_{1}\right)}{\left(x_{2}-x_{1}\right)\left(y_{2}-y_{1}\right)}\left(x_{2}-x\right)\left(y_{2}-y\right)+\frac{f\left(x_{2},y_{1}\right)}{\left(x_{2}-x_{1}\right)\left(y_{2}-y_{1}\right)}\left(x-x_{1}\right)\left(y_{2}-y\right)+\\ \; & \frac{f\left(x_{1},y_{2}\right)}{\left(x_{2}-x_{1}\right)\left(y_{2}-y_{1}\right)}\left(x_{2}-x\right)\left(y-y_{1}\right)+\frac{f\left(x_{2},y_{2}\right)}{\left(x_{2}-x_{1}\right)\left(y_{2}-y_{1}\right)}\left(x-x_{1}\right)\left(y-y_{1}\right) \end{array}, \end{array}$$

where $\left(x_{1},y_{1}\right)$, $\left(x_{1},y_{2}\right)$, $\left(x_{2},y_{1}\right)$, $\left(x_{2},y_{2}\right)$ are four known points, and the characteristics of the $P_{0}$-layer were obtained after bilinear interpolation as follows:

(3) $$\begin{array}{c} P_{0}=C_{2}+C_{3}f\left(x,y\right)+C_{4}f\left(x,y\right)+C_{5}f\left(x,y\right) \end{array}$$

The final generated feature layers were $P_{2}$, $P_{3}$, $P_{4}$, and $P_{5}$, which fully utilized the deep and shallow features by fusing these different layers, resulting in improved prediction performance.

**Figure 8:**
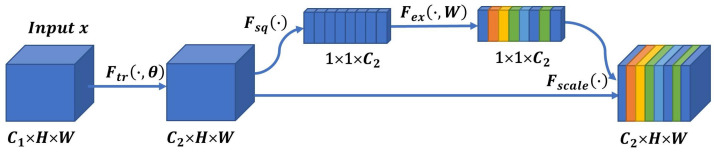
Channel attention mechanism.

Digestive endoscopy images are complex, and the presence of artifacts makes it difficult to distinguish between true targets. To enhance the expressive power of the features of images, a channel attention mechanism was employed in the FPN object detection model. For feature-mapping layers of different scales, the channel attention mechanism was used to capture the feature dependencies between different channel maps. This involves calculating the weighted feature vectors that represent the explicit correlation between the feature channels, as shown in Figure 8.

### 3.3. Loss Function

**Figure 9:**
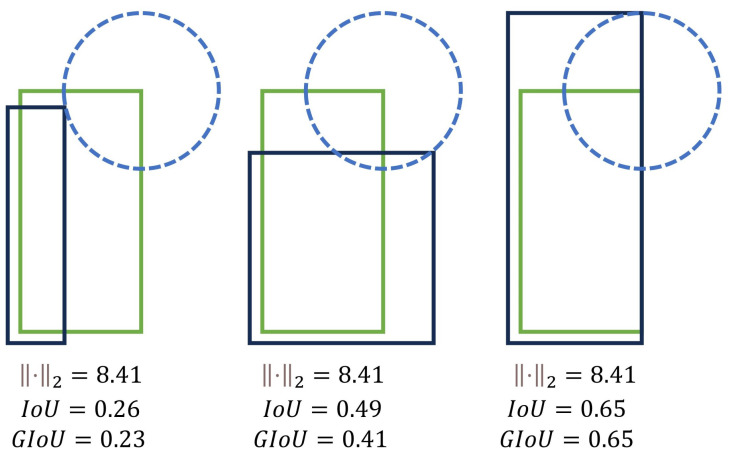
Three cases in L2 norm distance.

We introduced GIoU [22] as a new evaluation index loss function to replace the original evaluation index, IoU, in the cascade R-CNN. The GIoU formula ensures that the loss function for the target detection box regression is scale-invariant and maintains consistency between the optimization objective and the loss function. In the context of artifact identification in digestive endoscopy, there is a significant imbalance between positive and negative samples, which makes the training of the bounding box scores more challenging. Metrics based on L1 and L2 norms may yield significantly different IoU values for the two bounding boxes at the same distance. As shown in Figure 9, the example with the bounding boxes is represented by two corners. In all three cases, the distance between the representation of the two rectangles is the same for L2 norm; however, the IoU and GIoU values are very different. Furthermore, when IoU is the same, it only indicates that the Intersection over Union of the target box and the detection box is identical, but the actual size of the predicted box may be completely different. This highlights that neither IoU nor the L2 norm can adequately reflect the detection performance. Therefore, such loss functions are not ideal for predicting bounding boxes. Unlike IoU, which focuses only on the overlapping area, GIoU considers both overlapping and nonoverlapping regions, providing a better reflection of their intersection.

**Figure 10:**
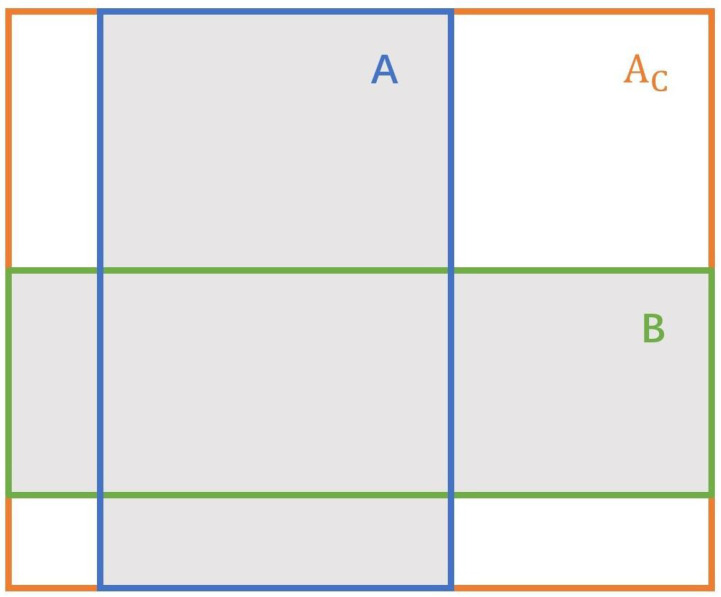
Generalized Intersection over Union diagram.

The introduction of the GIoU loss function has significant implications in the proposed method; it overcomes the limitations associated with traditional loss functions in the context of artifact detection. This loss function promotes a more precise bounding box regression and enhances the model’s ability to detect artifacts of varying sizes and shapes. This improvement augments the depth and accuracy of our research, thereby contributing to a more reliable analysis of endoscopic images, as shown in Figure 10.

The GIoU formula is:

(4) $$\begin{array}{c} GIoU=IoU-\frac{\left|A_{c}-U\right|}{\left|A_{c}\right|}, \end{array}$$

where *U* is the union of the prediction box and GT box, and $A_{c}$ is the minimum closure of the prediction box and real box.

### 3.4. Image Quality Score

Reading poor-quality medical images can significantly affect the efficiency of the diagnostic work. Therefore, based on the artifact detection results discussed in the previous section, we proposed a method to assess the quality of endoscopic images. This method aims to provide a visual indication of the effectiveness of an image. The image quality score (QS) is based on the (a) type, (b) region, (c) and location of the detected artifacts, and (d) the confidence of the detected artifacts. We improved the method based on Ali [3], where weights are assigned to each category, and the average weight is computed as the quality score.

Class weights $\left(w_{C}\right)$: artifact (0.50), specularity (0.10), saturation (0.10), blur (0.20), contrast (0.10), bubbles (0.02), instrument (0.02), and blood (0.02).

Area weight $\left(w_{A}\right)$: percentage of the total image area occupied by all detected artifact and normal areas.

Location weight $\left(w_{L}\right)$: center (0.5), left (0.25), right (0.25), top (0.25), bottom (0.25), top left (0.125), top right (0.125), bottom left (0.125), and bottom right (0.125).

The confidence weight $\left(w_{Co}\right)$ represents the confidence level of the detection results.

(5) $$\begin{array}{c} QS={\left\lfloor 1-\sum_{B}\left(\lambda_{A}W_{C}W_{Co}W_{A}+\lambda_{L}W_{C}W_{Co}W_{L}\right)\right\rfloor }_{0} \end{array}$$

**Figure 11:**
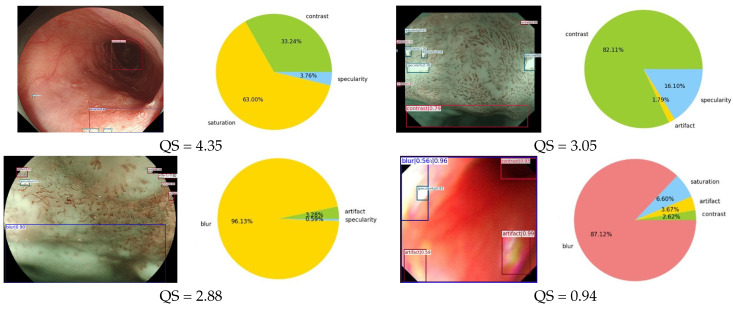
Quality assessment based on multiple parameters. The left side shows the artifact target detection results, and the right side shows the area ratio of different artifacts and the calculated mass score below.

To align with the numerical range of subjective ratings by experts and present the image assessment results in a more intuitive manner, this study mapped the QS values to a range of 0–5. The mapped results are presented in Figure 11.

## 4. Results and Discussion

### 4.1. Evaluation of Object Detection Results

The experimental setup in this study was as follows: Ubuntu 18.04 operating system, GeForce RTX 2060 graphics card, and Intel(R) Core(TM) i9-10940X processor. The improved cascade R-CNN network model proposed in this study was implemented within the mm detection framework version 2.25.0. The selected optimizer is a stochastic gradient descent with an initial momentum of 0.9. The total number of iterations was 5800. The training time for the first complete epoch of the model was 453 s, which gradually decreased over time. The total training time for 50 epochs was 18,537 s, 5.15 h.

This study employed commonly accepted evaluation metrics for object detection methods. Using a specific IoU threshold, true positives (TP), true negatives (TN), false positives (FP), and false negatives (FN) were defined as the classification outcomes. Furthermore, “all detections” refers to the total number of predicted bounding boxes, while “all ground truths” represents the total number of GT annotations.

(6) $$\begin{array}{c} \;\mathrm{Precision}\;=\frac{TP}{TP+FP}=\frac{TP}{\;\mathrm{all}\;\mathrm{detections}\;} \end{array}$$

(7) $$\begin{array}{c} \;\mathrm{Recall}\;=\frac{TP}{TP+FN}=\frac{TP}{\;\mathrm{all}\;\mathrm{GTs}\;} \end{array}$$

Average precision (AP) is a commonly used metric in object detection that evaluates model performance by calculating the area under the precision–recall (PR) curve. This reflected the AP at each unique recall level. AP is defined based on PASCAL VOC 2010 [23]:

(8) $$\begin{array}{c} AP=\sum_{r=i}^{n-1}\left(r_{i+1}-r_{i}\right)p_{\mathrm{interp}\;}\left(r_{i}+1\right) \end{array}$$

Through comparative experiments, we compared the performance of the proposed method with that of other networks in our dataset. From the AP values shown in Table 4 and Table 5, it can be seen that the proposed method achieved the best performance in all typical networks.

Through the implementation of ablative experiments, our primary objective was to investigate the performance characteristics of the network components. While keeping the other experimental conditions constant, we conducted separate experiments on the improved FPN and GIoU components using the proposed method. Based on the AP in Table 6, we observed that these components yielded improvements in the original model, thereby demonstrating the assisting role of the network components in learning pseudo-shadow features.

**Figure 12:**
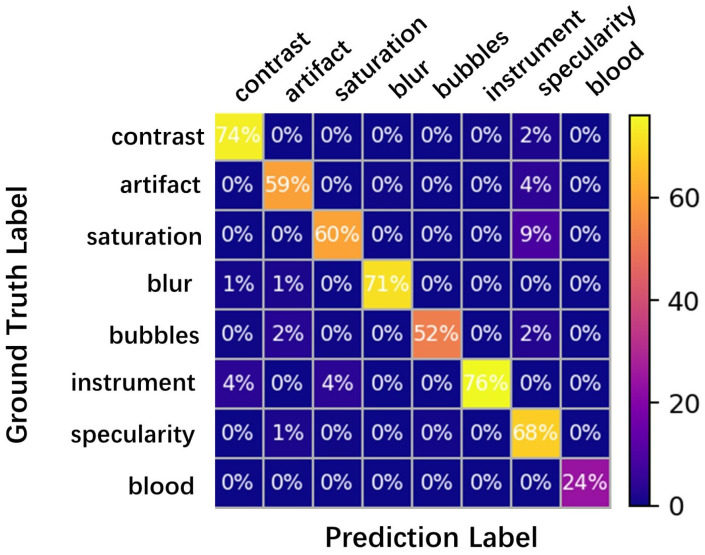
Confusion matrix showing true positive detections and confused classes in all eight classes.

The confusion matrix is an N×N matrix used to evaluate the performance of a classification model, where *N* represents the number of target categories. The matrix compares the actual and predicted class labels and assesses the predictive performance of the model. By analyzing the entire dataset, one can determine the number of correctly and incorrectly predicted samples, thus measuring the model’s predictive effectiveness. The confusion matrix, as presented in Figure 12, illustrates the performance for each class, with all classes except “blood” exhibiting performance exceeding 50%. This finding substantiates the existence of significant differences in the learned features among various classes. However, the “blood” class displays a slightly lower performance owing to the diverse morphological characteristics of blood within the gastrointestinal tract lacking distinct typical features.

The test results using the proposed method are presented in Table 7, using images from WLI, NBI, and iodine-staining modalities as examples. The table shows that the accurate detection of most defects was achieved in these three types of images. The detected bounding boxes effectively encompassed the abnormal regions without redundancy.

**Figure 13:**
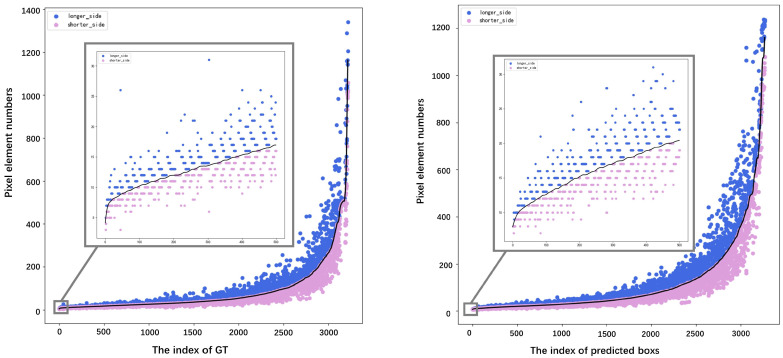
Square root of the area of the GT (**left**) and predicted box (**right**).

Figure 13 depicts the region-based statistical distributions of the GT (left) and predicted results (right) in the test dataset, arranged based on the region size. We observed that the predicted results exhibited a similar trend to those of the GT, indicating stable and reliable detection. This observation was further supported by specific evaluation metrics. In addition, the figure shows magnified views of the detection results for small targets, demonstrating the network’s ability to accurately capture defects with lengths smaller than 30 pixels.

### 4.2. Evaluation of the Quality Score

This study used a set of 100 randomly selected images with different quality ratings to evaluate the proposed quality assessment scheme based on the quality rankings provided by the three experts. Endoscopic images are different from natural images, and clinicians will randomly select the images of interest to shoot when performing image acquisition. There is no excellent reference image without distortion; therefore, the image quality assessment method without reference is used for image comparison. Four no-reference image quality assessment methods, NIQE [13], BRISQUE [6], MUSIQ [8], and NIMA [9], were compared with the method proposed in this paper.

Because the numerical values of the image evaluation scores represent only the quality levels of the images and lack practical significance, we employed the Spearman correlation coefficient to assess the monotonic relationship between the two variables. A larger value indicated a stronger correlation. Correlations were calculated using the following formula:

(9) $$\begin{array}{c} \rho_{S}=\frac{\sum_{i=1}^{N}\left(R_{i}-\bar{R}\right)\left(S_{i}-\bar{S}\right)}{{\left[\sum_{i=1}^{N}{\left(R_{i}-\bar{R}\right)}^{2}\sum_{i=1}^{N}{\left(S_{i}-\bar{S}\right)}^{2}\right]}^{\frac{1}{2}}} \end{array}$$

In Equation (9), *N* is the total number of observations, $R_{i}$ and $S_{i}$ are the grades of the observed value *i*. $\bar{R}$ and $\bar{S}$ are the average grades of the variables *x* and *y*, respectively.

A *p*-value test was used to determine the probability of the observed outcome occurring randomly.

Table 8 shows that the proposed quality score (QS) method has an average Spearman correlation coefficient of 60.71% with the rankings provided by the three experts. High correlation coefficients indicate a significant relationship between the proposed method and the quality scores suggested by the experts.

**Figure 14:**
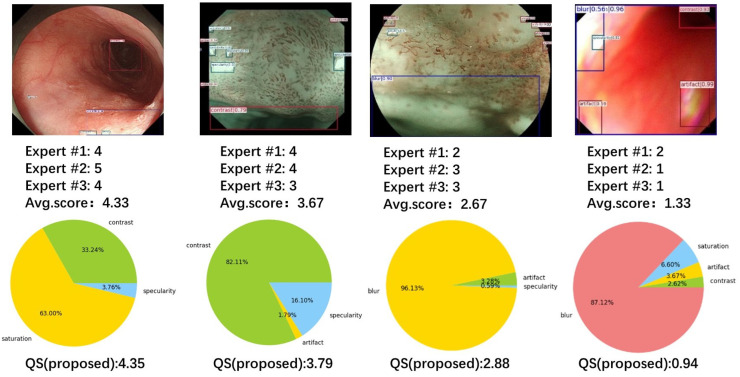
Artifact target detection results, scores from various experts, scores from the proposed method, and the proportion of detected artifact regions.

**Figure 15:**
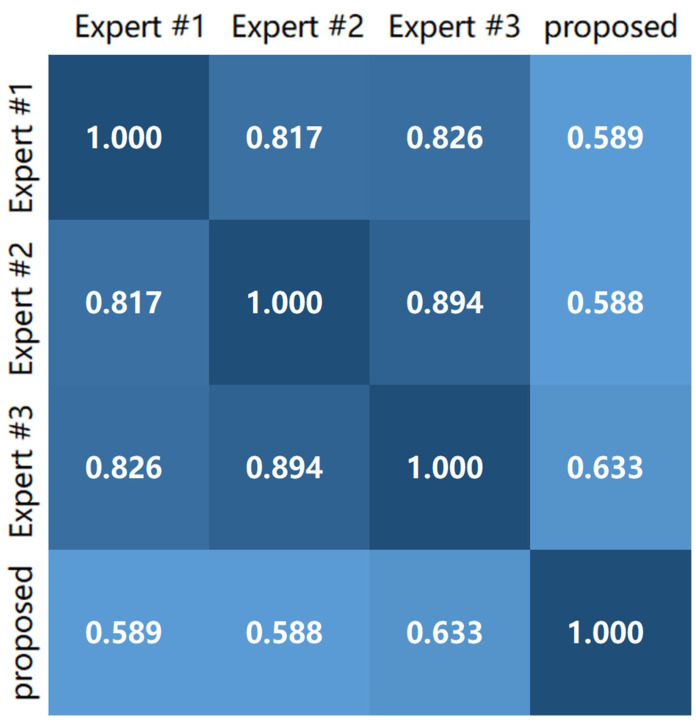
Heatmap between the mass scores of the three experts and the QS calculated by the proposed method.

Figure 14 presents four example images displaying the scores given by the three experts, the average score, and QS computed using the proposed method. The QS scores of the images of different qualities are close to the expert scores. Figure 15 illustrates the correlation between the scores determined by the three experts and the proposed method.

### 4.3. Discussion

The evaluation scores of the proposed quality assessment method for endoscopic images had a strong correlation with the expert ratings. This correlation was based on the category, confidence, area, and position information extracted from the artifact detection results. These findings indicate that the quality scores have a practical significance. The poor performance of existing no-reference image quality assessment methods, such as NIQE, BRISQUE, MUSIQ, and MANI, in evaluating digestive endoscopic images may be because these methods are primarily designed for natural images. This highlights the requirement for more specific approaches to evaluate the quality of digestive endoscopy images, considering their unique characteristics and environment. Owing to the tight integration of the proposed image quality assessment method with the object detection results, a strong interpretability was achieved, compared to the four methods mentioned above. In clinical applications, clinicians can rely on the quality scores of digestive endoscopic images as a primary quality control measure, thereby reducing the time required for the manual inspection of patient images.

The proposed object detection method is more sensitive to contrast, blur, instruments, and specularity, owing to the availability of sufficient data and features. However, the detection of blood objects showed a slightly lower performance owing to the limited training data and diverse morphologies of blood presentations. Future studies should focus on improving the detection of blood-related issues. Gastrointestinal endoscopy images encompass various modalities, and the proposed object detection method was validated on WLI, NBI, and iodine-stained modalities based on the imaging modalities offered by Olympus and Fujifilm instruments. However, because of the lack of data for linked color imaging and blue laser imaging modalities, the detection performance of these modalities requires further verification.

Based on the research presented in this paper, future work should focus on image restoration for images containing artifacts, building upon the foundation of object detection, which has a clinical significance. In future work, we will consider conducting comparative experiments for other versions of the YOLO series models. To enhance the robustness and generalizability of the conclusions, it is proposed to replicate the study in diverse geographical regions around the world. Such multilocation replication endeavors are necessary to validate the applicability and reproducibility of the methods and results across different contexts and populations. Validating this research in other parts of the world will significantly enhance the credibility of the research and provide solid support for clinical practice and applications.

## 5. Conclusions

This study proposed an optimized method for detecting endoscopy artifacts that provides intuitive information regarding the location and category of eight types of artifacts and numerically indicates image quality. The average precision (AP) results of object detection demonstrate significant improvements, compared to traditional object detection methods. The results of the correlation analysis indicate that the proposed method achieved better performance in the evaluation of gastrointestinal endoscopic image quality.

The results also revealed that an improved FPN structure is employed in the cascade R-CNN model. The improved FPN structure integrated the features from different layers, by effectively using both deep and shallow features. A channel attention mechanism was used to capture the feature dependencies between different channel maps in the feature extraction network. In addition, the GIoU metric was introduced as a new evaluation index loss function in the cascade R-CNN. The experimental results in Section 4.1 demonstrate that the AP value of the proposed model surpassed those of existing classical object detection models. Moreover, the model accurately detected small targets within a range of less than 30 pixels. Building on the object detection results, this study introduced a novel NR-IQA method for digestive tract images that uses composite weights. The experimental results in Section 4.2 were highly correlated with the expert ratings, indicating the practical significance of the quality scores.

## Figures and Tables

**Table 1:** Image modalities and digestive tract site statistics.

Mode	Train (Esophagus/ Stomach/Unknown)	Validation (Esophagus/ Stomach/Unknown)	Test (Esophagus/ Stomach/Unknown)
WLI	738/730/19	91/90/2	93/89/4
NBI	154/150/13	28/6/4	27/5/5
iodine-stained	17/21/0	4/5/0	4/4/0
Sum	1842 (909/901/32)	230 (123/101/6)	231 (124/98/9)
Total	2303 (1156/1100/47)		

**Table 2:** Artifact types and label statistics.

Types	Number of Images that Contain the Artifact	Number of Labels	Label Ratio (%)
Blood	86	112	1.15
Blur	418	433	4.44
Bubbles	303	788	8.08
Instrument	66	71	0.73
Specularity	776	4582	46.98
Contrast	819	910	9.33
Saturation	466	582	5.97
Artifacts	696	2276	23.33
Total		9754	100

**Table 3:** Endoscopic image quality assessment principles.

Quality Score	Scoring Principles
5	Endoscopic image is perfect, artifacts are negligible.
4	Endoscopic image exhibits minimal and small-sized artifacts, which do not interfere with the image.
3	Endoscopic image exhibits large-sized or numerous artifacts, which slightly interfere with the image.
2	Endoscopic image exhibits large-sized and numerous artifacts, which interfere with the image.
1	Endoscopic image exhibits large number of artifacts, which seriously interfere with the image.

**Table 4:** AP for typical networks.

Network	AP	${\mathrm{AP}}^{\mathrm{IoU}=0.50}$	${\mathrm{AP}}^{\mathrm{IoU}=0.75}$	${\mathrm{AP}}^{\mathrm{small}}$	${\mathrm{AP}}^{\mathrm{medium}}$	${\mathrm{AP}}^{\mathrm{large}}$
Cascade R-CNN [18]	0.149	0.335	0.102	0.031	0.109	0.186
Faster R-CNN [19]	0.203	0.395	0.178	0.033	0.144	0.266
PANet [24]	0.211	0.416	0.195	0.030	0.146	0.272
Sparse R-CNN [25]	0.136	0.278	0.120	0.018	0.083	0.175
YOLOV3 [26]	0.140	0.321	0.114	0.018	0.094	0.177
RetinaNet [27]	0.136	0.310	0.090	0.059	0.102	0.142
TridentNet [28]	0.324	0.581	0.301	0.140	0.216	0.338
YOLOV4 [17]	0.339	0.597	0.327	0.160	0.225	0.346
YOLOV5 [29]	0.346	0.608	0.330	0.164	0.239	0.358
**Proposed**	**0.370**	**0.624**	**0.361**	**0.171**	**0.250**	**0.392**

**Table 5:** AP for labels.

Network	Artifact	Blood	Blur	Bubbles	Contrast	Instrument	Saturation	Specularity
Cascade R-CNN	0.135	0.010	0.132	0.088	0.369	0.523	0.224	0.119
Faster R-CNN	0.146	0.021	0.187	0.083	0.367	0.522	0.221	0.129
PANet	0.150	0.028	0.217	0.083	0.349	0.534	0.226	0.130
Sparse R-CNN	0.086	0.002	0.115	0.024	0.284	0.379	0.148	0.051
YOLOV3	0.116	0.015	0.157	0.040	0.250	0.345	0.143	0.057
RetinaNet	0.154	0.001	0.370	0.055	0.220	0.002	0.118	0.168
TridentNet	0.294	0.011	0.537	0.200	0.392	0.524	0.306	0.332
YOLOV4	0.266	0.0618	0.542	0.208	0.363	0.597	0.255	0.330
YOLOV5	0.270	0.0749	0.549	0.221	0.378	**0.667**	0.278	0.332
**Proposed**	**0.318**	**0.105**	**0.608**	**0.242**	**0.410**	0.598	**0.333**	**0.342**

**Table 6:** Ablation experiments.

Experiments	Improved FPN	GIoU	AP
1	-	-	0.149
2	✓	-	0.301
3	-	✓	0.293
4	✓	✓	0.370

**Table 7:** Example of artifact detection using our network in different endoscopic modalities.

Mode	Artifact	Blood	Blur	Contrast	Instrument	Saturation	Specularity	Bubbles
WLI	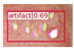	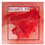	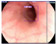	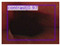	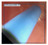	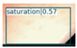	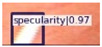	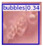
NBI	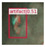	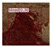	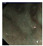	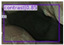	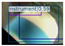	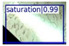	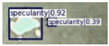	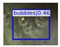
Iodine-stained	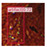	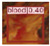	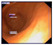	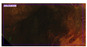	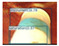	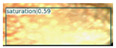	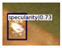	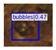

**Table 8:** Measured relevance using the correlation measure of the Spearman scale and its corresponding *p*-value.

IQA(NR)	Spearman Rank Correlation [−1,+1]					
	Corr.Value			* p * -Value		
	vs. Expert #1	vs. Expert #2	vs. Expert #3	vs. Expert #1	vs. Expert #2	vs. Expert #3
BRISQUE	0.1431	0.0403	0.0957	0.1532	0.6893	0.3411
NIQE	0.2389	0.3166	0.2674	0.0161	0.0013	0.007
MUSIQ	0.2240	0.2565	0.2804	0.0243	0.0096	0.0045
NIMA	0.0722	0.0025	0.0556	0.4727	0.9801	0.5811
**Proposed**	**0.5894**	**0.5986**	**0.6334**	**5.09 × 10${}^{-10}$**	**5.49 × 10${}^{-10}$**	**9.47 × 10${}^{-12}$**

## Data Availability

The data that support the findings of this study are available from the corresponding author, upon reasonable request.
